# Orbital decompression with intraorbital corticosteroid injection during the active phase of thyroid eye disease: outcomes in a real-world cohort

**DOI:** 10.3389/fopht.2026.1935053

**Published:** 2026-09-29

**Authors:** Adam J. Neuhouser, Sarah Skender, Jane Z. Spadaro, Alon Kahana

**Affiliations:** 1 Kahana Oculoplastic and Orbital Surgery, Livonia, MI, United States; 2 Department of Ophthalmology, Corewell Health William Beaumont University Hospital Eye Institute, Royal Oak, MI, United States; 3 Department of Ophthalmology, Oakland University William Beaumont School of Medicine, Rochester, MI, United States

**Keywords:** Clinical Activity Score, dysthyroid optic neuropathy, graves orbitopathy, intraorbital corticosteroid, orbital congestion, orbital decompression, thyroid eye disease

## Abstract

**Background:**

Orbital decompression is an effective surgery for reducing orbital congestion and proptosis for patients with thyroid eye disease (TED). However, it is commonly stated that elective orbital decompression, i.e., for reasons other than compressive optic neuropathy, should await the quiescent phase of the disease. The reason is to avoid inducing a flare-up of the orbitopathy secondary to surgical inflammation. Still, this means that patients with moderate to severe TED who are suffering from symptoms associated with TED must wait months to years before initiating surgical treatment. The purpose of this study is to evaluate the effectiveness of orbital apex decompression combined with intraorbital corticosteroid injection in patients with active TED, defined as Clinical Activity Score (CAS) ≥3.

**Methods:**

This retrospective, single-center cohort study included patients with active TED (pre-operative CAS ≥ 3) who underwent single or staged orbital decompression between February 2023 and September 2025. All lateral wall decompressions included an intraorbital injection of a combination triamcinolone and dexamethasone as part of the operative technique. Primary outcomes included change in CAS, change in proptosis, and postoperative disease progression (≥ 2-point increase in CAS at ≥ 8 weeks). Secondary analysis compared outcomes in patients with and without perioperative treatment using disease-modifying therapy (DMT).

**Results:**

Thirty-six patients met inclusion criteria; 14 had preoperative dysthyroid optic neuropathy. Mean CAS decreased from 4.7 to 1.6 (Δ –3.1; 95% CI –3.92 to –2.36), and mean proptosis decreased from 22.7 mm to 19.7 mm (Δ –3.0 mm; 95% CI –3.28 to –2.23). One patient with severe TED met criteria for postoperative disease progression. Outcomes were consistent across subgroups regardless of perioperative DMT exposure. No patients developed new-onset diplopia.

**Conclusions:**

Orbital decompression combined with intraorbital corticosteroid injection during active TED was associated with clinically meaningful reductions in CAS and proptosis, with infrequent postoperative deterioration and no major surgical complications. These findings provide real-world evidence supporting use of this combined approach during active disease, including in patients without dysthyroid optic neuropathy. These results are specific to a particular surgical technique and to the perioperative management of underlying thyroid status and risk factors associated with TED exacerbation.

## Introduction

Thyroid eye disease (TED) is an autoimmune inflammatory orbitopathy most commonly associated with hyperthyroidism. A complex interplay of immune cells and cytokines stimulates orbital fibroblasts and adipocytes through thyroid-stimulating hormone receptor (TSHR) and insulin-like growth factor-1 receptor (IGF-1R) signaling, leading to glycosaminoglycan deposition, adipogenesis, and tissue fibrosis (1–3). This produces the classic findings of proptosis, eyelid retraction, extraocular muscle enlargement, restrictive strabismus, exposure keratopathy, and, in severe cases, compressive optic neuropathy (2).

Traditionally, TED has been described as a biphasic disease—an active inflammatory phase lasting one to three years, followed by an inactive fibrotic phase (4). However, contemporary evidence suggests that TED is a heterogeneous, relapsing-remitting autoimmune condition, with recurrence rates ranging between 5–16% following diagnosis (5), and can be influenced by modifiable risk factors including elevated thyroid-stimulating immunoglobulin (TSI) levels, thyroid dysfunction, smoking, exposure to radioactive iodine, vitamin D deficiency, and dyslipidemia (6–11).

Medical management of moderate to severe active TED centers on systemic corticosteroids and/or IGF-1R inhibitors, as supported by international consensus guidelines (12, 13). Surgical intervention is often required for persistently symptomatic or vision-threatening disease, such as dysthyroid optic neuropathy or exposure keratopathy with resultant corneal breakdown (3, 14–16). Orbital decompression is typically deferred until the inactive phase to minimize the perceived risk of postoperative disease progression. Conventional teaching postulates that surgery during the phase of active inflammation will exacerbate disease activity (17); however, there are limited comparative studies in the current literature supporting this claim. Reported rates of early postoperative progression following decompression in active TED range from 1% to 7%, with time to progression ranging up to 36 weeks following surgery (5, 18–20).

Emerging data demonstrate that early decompression in select patients—particularly those with dysthyroid optic neuropathy—can reduce congestion, relieve pain and pressure, and potentially hasten resolution of orbital inflammation by restoring venous outflow and lymphatic drainage (11, 21–23). The present study evaluates outcomes of apical orbital decompression combined with intraorbital corticosteroid injection performed during the active phase of TED (Clinical Activity Score (CAS) ≥ 3). Using real-world data, we assess whether this combined approach is safe and associated with meaningful improvement beyond the traditional indication of optic neuropathy—specifically for patients presenting with retrobulbar pain and/or pain with eye movement, disfiguring proptosis, and/or exposure keratopathy.

## Methods

This retrospective, single-center cohort evaluated patients with active TED (CAS ≥ 3) who underwent orbital decompression by the senior author between February 2023 and September 2025. This study was approved by the Institutional Review Board of Corewell Health William Beaumont University Hospital and adhered to the tenets of the Declaration of Helsinki. The study was conducted in compliance with the Health Insurance Portability and Accountability Act (HIPAA).

Patients were included if they had a diagnosis of TED, a pre-operative CAS ≥ 3, and underwent any form of single or staged orbital decompression. Patients were excluded if they had a CAS ≤ 2, a follow-up shorter than three months, or incomplete data. Demographics (age, sex, ethnicity), smoking status, thyroid status categorized as Graves hyperthyroidism, euthyroid, or hypothyroid Graves disease based on clinical endocrinology documentation, treatments, pre- and postoperative CAS, Hertel exophthalmometry, and intraocular pressure (IOP) were extracted from the electronic health record. Diplopia was assessed by patient report and motility examination at baseline and last follow-up; formal quantification with binocular visual field testing or Gorman scoring was not performed, as this was not a primary strabismus study.

In unilateral cases, operative-eye Hertel values were used; in bilateral cases, the mean of both eyes was recorded to avoid pseudoreplication. Operative reports were reviewed to document surgical indication (retrobulbar pain or pain with eye movement; proptosis with exposure keratopathy; dysthyroid optic neuropathy), wall(s) decompressed (lateral, medial, multiwall), and whether surgery was unilateral or staged bilateral. Exposure to disease-modifying therapies (DMTs) before and after surgery was recorded. The preoperative thyroid-stimulating hormone (TSH) was recorded from the most recent laboratory testing prior to decompression surgery.

The primary endpoints were change in CAS, change in proptosis (Hertel, mm), and presence of postoperative worsening (≥ 2-point CAS increase at ≥ 8 weeks postoperatively and before any postoperative DMT). Secondary endpoints examined whether improvement in disease activity was associated with perioperative DMT exposure. A *post hoc* analysis was also performed for patients whose primary surgical indication was retrobulbar pain and/or pain with eye movement. Disease-modifying therapy was defined as exposure to therapeutic-strength corticosteroids (intravenous methylprednisolone, oral prednisone, or intraorbital injection of 1:1 mix of triamcinolone 40 mg/mL and dexamethasone 4 mg/mL) or an IGF-1R inhibitor (teprotumumab or veligrotug administered within a clinical trial).

Preoperative exposure to DMT was defined as exposure to therapeutic-strength corticosteroid within 3 months of surgery, or to an IGF-1R inhibitor within 12 months. Postoperative exposure to any DMT was documented and separated into an early (< 8-week) versus late (≥ 8-week) postoperative exposure.

Endpoint CAS was defined as the last recorded score at ≥ 8 weeks after decompression to allow resolution of postoperative edema or the final CAS immediately prior to initiation of any postoperative DMT, thereby avoiding artificially low scores attributable to medical therapy. If postoperative steroids were administered, and ≥ 12 weeks had elapsed by final follow-up, the CAS at last follow-up was accepted to represent the surgical effect, as this served as a sufficient washout period. For patients who received an IGF-1R inhibitor post-op, the CAS immediately prior to initiation was used.

The surgical techniques focused on apical decompression, with contouring of the deep sphenoid in lateral decompression, and opening of the posterior ethmoid along with removal of the orbital process of the palatine bone in medial decompression. Bone removal was performed using ultrasonic aspiration (Sonopet, Stryker, Kalamazoo, MI) and stereotactic navigation. During lateral wall decompression, intraconal fat was usually excised (median 1.25 mL, range 0 mL to 3 mL, mean 1.2 mL) and 1 mL of 1:1 triamcinolone/dexamethasone was injected into the lacrimal gland and surrounding fat as a standard component of the operative technique. This intraorbital injection serves as an integral element of the treatment algorithm and is expected to contribute to early anti-inflammatory and anti-congestive effects alongside the mechanical decompressive effect. The primary goal of each decompression surgery was to expand the volume of the orbital apex.

Statistical analysis was performed with the Numiqo Statistics Calculator (Numiqo, Boston, MA). Quantitative variables are reported as means ± SD; changes are presented as mean differences with 95% confidence intervals (CIs). Because this is a real-world descriptive study, statistical interpretation was based on the 95% CI rather than formal hypothesis testing; differences were considered clinically meaningful when the CI did not cross zero.

## Results

A total of 36 patients met inclusion criteria. The mean follow-up duration was 12.1 months from the date of surgical decompression. Baseline characteristics are summarized in **Table 1**. The mean age was 58.6 ± 10.9 years; 78% were female. Most patients had hyperthyroid Graves disease (94%), and 50% were former smokers. Preoperative TSH levels (n = 36) demonstrated a mean of 1.84 ± 3.08 mIU/L and a median of 0.71 mIU/L (range, 0.01–17.5 mIU/L), with the majority of values within or near the reference range. Indications for decompression included dysthyroid optic neuropathy (39%), retrobulbar pain and/or pain with eye movement (33%), and disfiguring proptosis with exposure keratopathy (28%). Surgical approaches reflected real-world practice: 42% underwent staged bilateral lateral wall decompression, 33% underwent staged bilateral lateral + medial, and 13.9% staged unilateral lateral + medial wall decompression. Mean estimated intraoperative blood loss was 54.5 mL per procedure (range, 5—520 mL).

**Table 1:** Baseline characteristics of patients with active thyroid eye disease (CAS ≥ 3).

Characteristic (n = 36)	Value
Age (mean ± SD)	58.6 ± 10.9 years
Sex, n (%)	
Male	8 (22.2%)
Female	28 (77.8%)
Ethnicity, n (%)	
Caucasian	30 (83.8%)
African American	4 (11.1%)
Asian	1 (2.8%)
Other	1 (2.8%)
Smoking Status, n (%)	
Active	6 (16.7%)
Former	18 (50%)
Never	12 (33.3%)
Underlying Thyroid Disorder, n (%)	
Hyperthyroid Graves	34 (94.4%)
Euthyroid	2 (5.6%)
Hypothyroid Graves	0
Preoperative TSH, mIU/L	
Mean ± SD	1.84 ± 3.08*
Median (range)	0.71 (0.01–17.5)
Surgical Indication, n (%)	
Retrobulbar pain/Pain with eye movement	12 (33.3%)
Disfiguring proptosis and exposure keratopathy	10 (27.8%)
Dysthyroid optic neuropathy	14 (38.9%)
Decompression Surgery Performed, n (%)	
Unilateral lateral wall	3 (8.3%)
Staged unilateral lateral + medial wall	5 (13.9%)
Staged bilateral lateral wall	15 (41.7%)
Staged bilateral lateral + medial wall	12 (33.3%)
Simultaneous bilateral medial wall	1 (2.8%)
Pre-op Disease Modifying Therapy Exposure, n (%)	
IGF-1R inhibitor ≤ 12 months pre-op	3 (8.3%)
Steroid** exposure ≤ 3 months pre-op	4 (11.1%)
Post-op Disease Modifying Therapy Exposure, n (%)	
IGF-1R inhibitor post-op	3 (8.3%)
Steroid** exposure post-op	6 (16.7%)
Follow-up duration, mean	12.1 months

CAS, Clinical Activity Score; SD, standard deviation; IGF-1R, insulin-like growth factor-1 receptor; pre-op, preoperative; post-op, postoperative; TSH, thyroid-stimulating hormone.

*Two patients had markedly elevated TSH values (6.24 and 17.5 mIU/L). Excluding these values, the mean TSH was 1.25 ± 1.31 mIU/L.

**Intravenous methylprednisolone, oral prednisone, or intraorbital injection of triamcinolone 40 mg/mL combined with dexamethasone 4 mg/mL (1:1 dilution).

Preoperative DMT exposure included teprotumumab in 3 patients (≤ 12 months) and therapeutic-strength steroids in 4 (≤ 3 months). Postoperative DMT included IGF-1R inhibitors (n = 3; 2 teprotumumab, 1 veligrotug administered as part of a clinical trial) and steroids (n = 6) (**Table 1**).

Among all patients (with endpoint CAS handling as defined in Methods), CAS decreased from 4.7 to 1.6 (Δ = –3.1; 95% CI, –3.92 to –2.36) and proptosis decreased from 22.7 mm to 19.7 mm (Δ = –3.0 mm; 95% CI, –3.28 to –2.23) (**Table 2**). One patient (2.8%; 95% CI, 0.5–14.3%) met criteria for postoperative worsening. Individual patient-level changes in CAS and proptosis are shown in **Figures 1**, **2**.

**Table 2:** Surgical outcomes following orbital decompression in active thyroid eye disease.

Primary Outcomes				
n = 36	Pre-op	Final	Change (Δ)	95% CI
CAS, mean	4.7	1.6	−3.1	(−3.92 to −2.36)
Proptosis (mm)	22.7	19.7	−3.0	(−3.28 to −2.23)
Postoperative worsening: ≥ 2-point CAS increase			1/36 (2.8%)	(0.5–14.3%)

CAS, Clinical Activity Score; CI, confidence interval; Δ, change from preoperative to final follow-up. Proptosis measured in millimeters by Hertel exophthalmometry. Postoperative worsening defined as a ≥2-point increase in CAS at ≥8 weeks following decompression.

**Figure 1:**
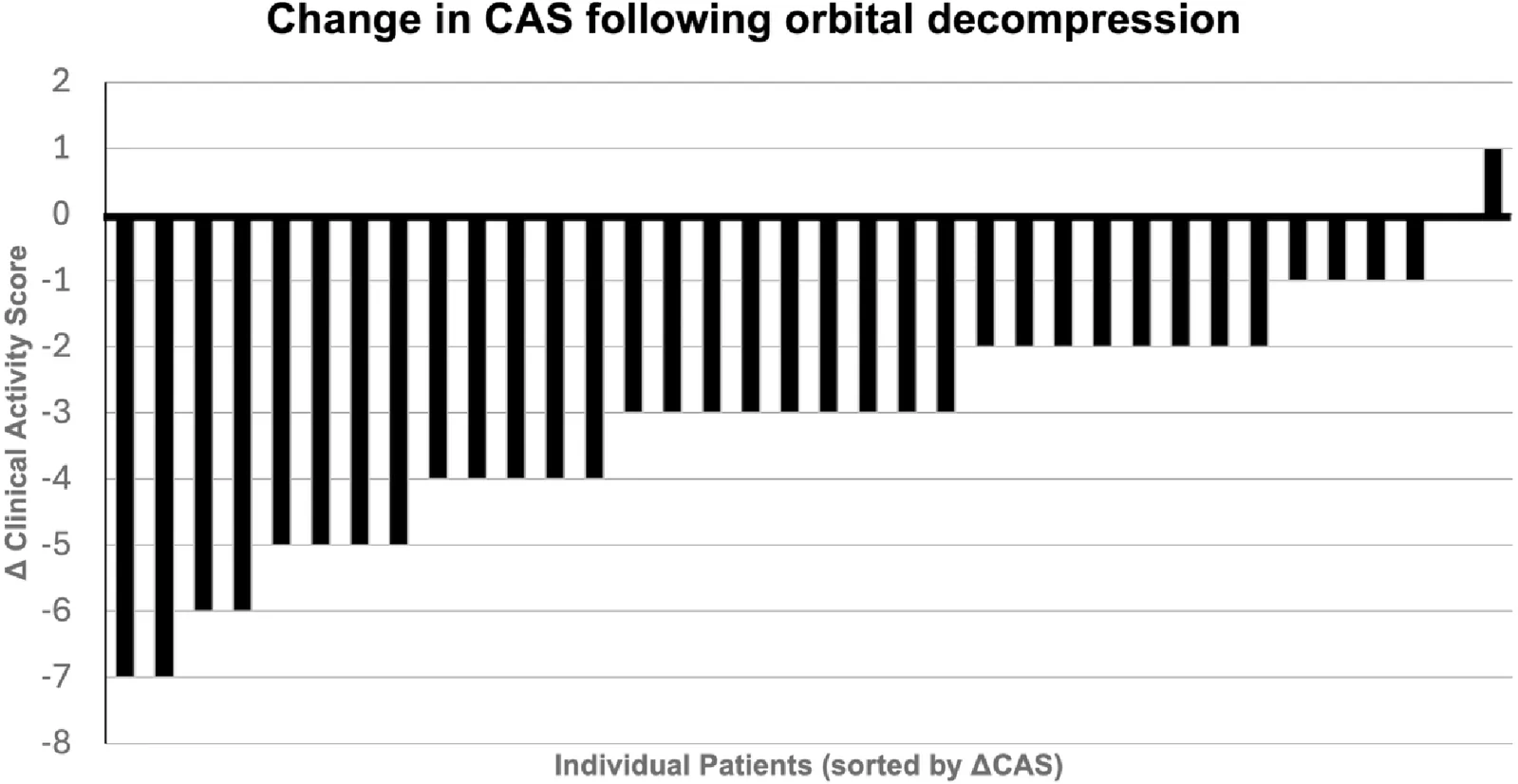
Change in Clinical Activity Score following orbital decompression. Change in Clinical Activity Score (ΔCAS) for individual patients following orbital decompression combined with intraorbital corticosteroid injection. Patients are ordered by magnitude of CAS change. Asterisk denotes the single patient meeting criteria for postoperative worsening.

**Figure 2:**
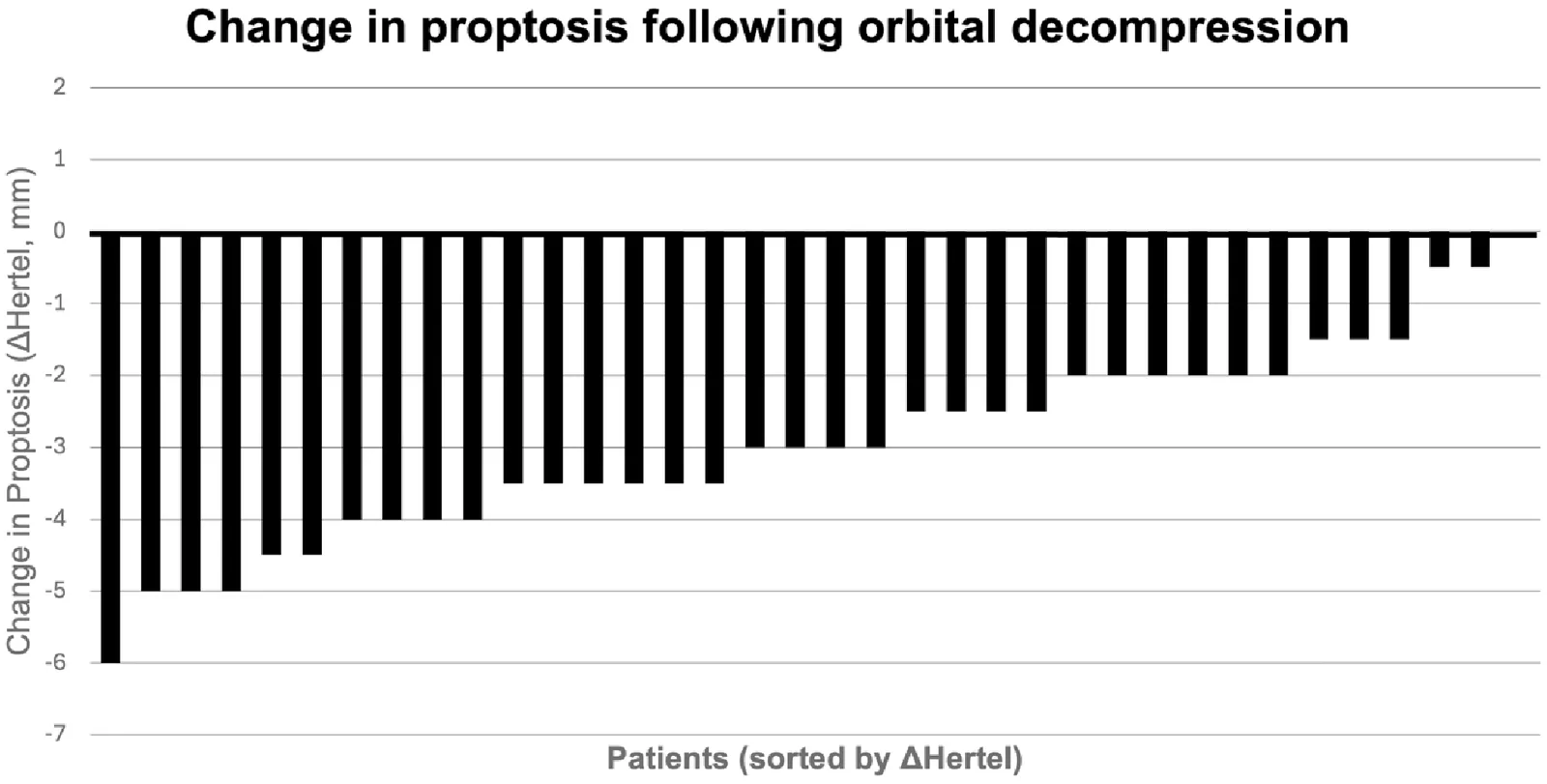
Change in proptosis following orbital decompression. Change in proptosis (ΔHertel, mm) for individual patients following orbital decompression. Patients are ordered by magnitude of proptosis reduction.

Diplopia was present preoperatively in 27 of 36 patients (75%). Following decompression, diplopia resolved with surgery alone in 4 patients (14.8% of those with preoperative diplopia). Of the remaining 23 patients with persistent diplopia, 18 achieved successful management with prism correction and/or strabismus surgery by last follow-up. Five patients had residual diplopia limited to extreme gaze and elected to defer prism or strabismus surgery given minimal functional impact. No patients developed new-onset diplopia following decompression.

Nine patients (25%) required DMT post-op (**Table 3**): 2 early (< 8 weeks) and 7 late (≥ 8 weeks). Of the two patients who required early DMT escalation, one received a single dose of intravenous methylprednisolone 500 mg for worsening periorbital edema, erythema, and chemosis in the early postoperative period. The second received a short oral prednisone taper (40 mg for 5 days, 20 mg for 5 days, then 10 mg for 5 days) for slight worsening of strabismus following left lateral wall decompression. In both patients, CAS had improved from baseline prior to escalation (mean ΔCAS to escalation: −2), and neither met criteria for postoperative worsening. The eight-week post-operative period was chosen to distinguish early postoperative inflammation from true disease progression. In both subgroups, CAS had improved before escalation (mean ΔCAS to escalation –2 in early, –1 in late). Only one patient met postoperative worsening criteria (≥ 2-point CAS increase at ≥ 8 weeks) and required DMT.

**Table 3:** Post-decompression disease-modifying therapy: early escalation (< 8 weeks) versus late progression (≥ 8 weeks).

Post-decompression disease-modifying therapy—early vs late		
Early post-op disease modifying therapy (< 8 weeks)		
	Value	95% CI
Patient with DMT < 8 weeks, n/N (%)	2/36 (5.6%)	(1.5–18.1%)
Δ CAS from pre-op to DMT visit, mean	−2	
≥ 2-point CAS increase before DMT	0/2	
Breakdown of escalation type, n/N:		
IV methylprednisolone 500 mg	1/2	
Oral prednisone	1/2	
Intraorbital steroid injection	0	
IGF-1R inhibitor	0	
Investigational drug	0	
Late Post-Op Disease Modifying Therapy (≥ 8 weeks)		
Patients with DMT ≥ 8 weeks, n/N (%)	7/36 (19.4%)	(9.8–35%)
Δ CAS from pre-op to DMT visit, mean	−1	
≥ 2-point CAS increase before DMT	1/7	
Breakdown of escalation type:		
IV corticosteroid	0	
Intraorbital steroid injection	4	
Oral prednisone	0	
IGF-1R inhibitor	2	
Investigational drug	1	

DMT, disease-modifying therapy; CAS, Clinical Activity Score; IGF-1R, insulin-like growth factor–1 receptor. Early escalation defined as initiation of disease-modifying therapy within 8 weeks following decompression; late progression defined as initiation ≥8 weeks postoperatively.

**Table 4** compares outcomes by preoperative DMT exposure. Patients without pre-op DMT (n = 29) had ΔCAS –2.8 and ΔHertel –3.2 mm; those with pre-op DMT (n = 7) had ΔCAS –2.7 and ΔHertel –2.2 mm. Differences were not significant (ΔCAS difference 0.1, 95% CI –2.73 to 2.95; ΔHertel difference 1.0 mm, 95% CI –0.17 to 2.03). The one patient who experienced postoperative worsening was in the preoperative DMT group. Outcomes were comparable regardless of prior medical therapy.

**Table 4:** Impact of pre-operative disease-modifying therapy on surgical outcomes.

Impact of pre-operative disease-modifying therapy on surgical outcomes				
	No Pre-op DMT (n = 29)	Pre-op DMT (n = 7)	Difference	95% CI
Δ CAS	−2.8	−2.7	0.1	(−2.73 to 2.95)
Δ Proptosis (mm)	−3.2	−2.2	1.0	(−0.17 to 2.03)
Postoperative worsening ≥ 2-point CAS	0/29	1/7		

DMT, disease-modifying therapy; CAS, Clinical Activity Score; CI, confidence interval; Δ, change from preoperative to final follow-up.

A *post hoc* subgroup analysis was performed in the 12 patients whose primary surgical indication was retrobulbar pain and/or pain with eye movement (**Table 5**). Mean CAS decreased from 4.9 preoperatively to 1.7 at the endpoint assessment (Δ −3.3; 95% CI, −4.83 to −1.67), while mean proptosis decreased from 22.4 mm to 19.6 mm (Δ −2.8 mm; 95% CI, −3.59 to −2.00). None of the 12 patients met criteria for postoperative worsening, developed new-onset diplopia, or experienced a major surgical complication. Five patients (41.7%) received postoperative DMT.

**Table 5:** Outcomes among patients undergoing decompression for retrobulbar pain and/or pain with eye movement (n = 12).

Outcomes in the pain/pressure subgroup (n = 12)				
n = 12	Pre-op	Endpoint	Change (Δ)	95% CI
CAS, mean	4.9	1.7	−3.3	(−4.83 to −1.67)
Proptosis (mm)	22.4	19.6	−2.8	(−3.59 to −2.00)
Postoperative worsening: ≥ 2-point CAS increase			0/12	
Postoperative DMT, n/N (%)			5/12 (41.7%)	
New-onset diplopia			0/12	
Major surgical complications			0/12	

CAS, Clinical Activity Score; CI, confidence interval; DMT, disease-modifying therapy; Δ, change from preoperative to endpoint measurement. Proptosis measured in millimeters by Hertel exophthalmometry. Postoperative worsening defined as a ≥2-point increase in CAS at ≥8 weeks following decompression and before initiation of postoperative disease-modifying therapy.

No patients suffered any significant intraoperative complication or post-operative adverse event related to orbital decompression surgery, such as postoperative vision loss, diplopia, hemorrhage, cerebrospinal fluid leak, sinusitis, or infection.

## Discussion

Orbital decompression has traditionally been reserved for patients in the inactive phase of TED, based on concerns that surgical manipulation during active inflammation could provoke disease exacerbation and lead to unpredictable postoperative results (14–16). This approach has led to the practice of treating active TED with corticosteroids or IGF-1R inhibitors while waiting for chronicity before surgery. Newer insights, however, challenge this sequence. Active TED involves significant orbital congestion from fat and muscle expansion, not just inflammation. Congestion may cause or exacerbate tissue hypoxia, which contributes to fibrosis (24). Therefore, decompression may benefit patients even in the active phase by improving venous outflow, reducing associated symptoms such as orbital pressure, and possibly reducing fibrosis. The critical technical requirement is that such surgical decompression target the orbital apex, the primary site of venous outflow.

In this real-world cohort, apical orbital decompression combined with a standardized intraorbital corticosteroid injection was associated with consistent, clinically meaningful improvements in both CAS and proptosis. These findings support reconsideration of the traditional timing of surgical intervention in active TED, though prospective data will ultimately be needed to establish causality.

An important aspect of our treatment protocol that distinguishes this cohort from prior series is the universal administration of an intraorbital 1:1 triamcinolone/dexamethasone injection at the time of lateral wall decompression. We consider this injection an integral component of our surgical technique, based on the rationale that anti-inflammatory support at the surgical site complements the mechanical decompressive effect. The combined approach targets both the congestive and inflammatory components of active TED simultaneously. Prior work by Eid et al. has demonstrated efficacy of orbital corticosteroid injection in active TED (15).

Our results align with the concept that decompression relieves congestive physiology—by improving venous outflow, reducing orbital pressure, and thereby diminishing inflammatory signs that contribute to CAS. Prior series in dysthyroid optic neuropathy have reported improvements in inflammatory parameters after urgent decompression (11, 21–23). The present cohort adds real-world outcome data for a standardized apical orbital decompression and intraorbital corticosteroid strategy during active disease, and includes clinical experience in surgical indications beyond compressive optic neuropathy. The 12 patients undergoing decompression primarily for retrobulbar pain and/or pain with eye movement also demonstrated reductions in CAS and proptosis without postoperative worsening (**Table 5**). Although this subgroup is small, these findings support further study of orbital decompression for these symptoms during active TED.

As contemporary TED management frequently includes corticosteroids and IGF-1R inhibitors, we prespecified endpoint CAS rules (≥ 8-week minimum; pre-DMT values; steroid washout ≥ 12 weeks) to minimize the contribution of medical therapy to endpoint CAS. Across subgroups, outcomes were consistent: ΔCAS and ΔHertel improvements were similar regardless of preoperative DMT exposure (**Table 4**). Postoperative DMT was administered in 25% of our patients; only two patients required DMT within 8 weeks, and neither met postoperative worsening criteria. In both early and late escalation groups, CAS had improved before DMT, supporting that the combined decompression-injection approach delivered clinical benefit even when adjunctive therapy was later necessary (**Table 3**).

The single patient meeting postoperative worsening criteria was a 73-year-old woman with severe, recurrent TED. She originally experienced TED symptoms at the time of her hyperthyroid diagnosis in the 1960s, and developed a TED episode in 2022 in the setting of elevated TSI (16.2 IU/L). Her preoperative TSH was 3.39 mIU/L. She required sequential treatment with intraorbital steroids, intravenous methylprednisolone, and was stabilized following a partial course of teprotumumab. She developed a recurrent flare-up of TED in 2024, 11 months following her last teprotumumab infusion. Clinical exam demonstrated CAS of 4 and compressive optic neuropathy, and bloodwork revealed elevated TSI (4.77 IU/L) and TRAB (4.02 IU/L). She underwent combined lateral and medial wall orbital decompression. By postoperative month five, her CAS had decreased to 1 with full symptom resolution. At postoperative month eight, she developed a third TED flare-up. Her CAS increased to 7, coinciding with a TSH elevation to 5.54 mIU/L, and was associated with abnormal thyroid antibody titers (TSI 9.65 IU/L, TRAB 9.45 IU/L); she received an intraorbital steroid injection which improved her symptoms. Given the delayed timing (8 months) and concurrent thyroid dysfunction, this episode is more likely attributable to persistent autoimmunity and systemic thyroid hormone level instability rather than a response to the decompression surgery.

These data support the hypothesis that early apical decompression combined with intraorbital corticosteroid injection is a reasonable option for patients with active TED who present with retrobulbar pain/pressure symptoms, debilitating diplopia requiring strabismus surgery (which should be preceded by orbital decompression) and/or disfiguring proptosis with exposure keratopathy — even in the absence of optic neuropathy. Our data presume management of disease modifiable risk factors (i.e. thyroid status, smoking, vitamin D deficiency, dyslipidemia), as our study population had a baseline mean TSH of 1.25 mIU/L, indicating a generally well-controlled thyroid status in the cohort at the time of surgery. The low rate of postoperative worsening over a relatively long follow-up duration (12.1 months), coupled with meaningful ΔCAS and ΔHertel reductions, underscores the relative safety of this approach. Importantly, decompression was not associated with new-onset diplopia, and many patients actually experienced improvement in their diplopia symptoms.

Based on our experience, we postulate that the traditional caution against orbital decompression in active orbitopathy originated in the era of anterior orbital decompression techniques that failed to adequately address venous outflow at the orbital apex. In that context, postoperative edema and inflammation can compound preexisting orbital congestion, leading to poor outcomes. Modern techniques focusing on contouring the deep sphenoid diploe, posterior ethmoid sinus, and removing the orbital process of the palatine bone – all at the orbital apex – are expected to meaningfully improve venous outflow and reduce congestion, making the surgery safer to perform in patients with elevated CAS.

Intraoperative bleeding in this series arose predominantly from perforating diploic venous channels within the greater wing of the sphenoid rather than from inflamed orbital soft tissue, which is encountered only in the final stages of lateral wall decompression when the periorbita is opened. During medial wall decompression, use of preoperative oxymetazoline nasal spray, early cauterization of the anterior ethmoidal artery, and stripping of ethmoid sinus mucosa, limited intra- and post-operative bleeding. We did not observe active inflammation to be a meaningful contributor to operative blood loss or to play a significant role in the technical difficulty of the surgery.

This study has several limitations inherent to its retrospective design. The sample size was modest, and the cohort is single-center, limiting generalizability. The absence of an untreated, injection-only, or inactive-phase control group prevents determination of whether the observed improvements resulted from decompression, intraorbital corticosteroid injection, patient selection, or the natural course of TED. The findings therefore apply to the combined intervention. The universal administration of an intraorbital corticosteroid injection as part of the operative technique means that the individual contributions of the mechanical decompression and the corticosteroid cannot be disentangled within this dataset; the outcomes reported reflect the combined intervention. Additionally, CAS is a measure that captures both inflammatory and congestive signs; it is possible that a subset of our patients had predominantly congestive rather than immunologically active disease, which may have favorably influenced CAS response regardless of intervention. Future studies using complementary measures of disease activity—such as orbital imaging, inflammatory biomarkers, or quality-of-life instruments—would help delineate the relative contributions of these components. Randomized prospective surgical trials are not feasible in this clinical context. The evidence base for orbital surgical practice must therefore rely on an accumulation of well-characterized retrospective cohort studies to inform treatment algorithms. Fourteen of 36 patients underwent decompression for dysthyroid optic neuropathy, and 22 underwent surgery for an alternative indication such as severe exophthalmos, restrictive strabismus, and/or orbital pain. Although all patients were included in the assessment of outcomes following decompression and intraorbital corticosteroid injection during active disease, the study was not designed to establish effectiveness for individual non-dysthyroid optic neuropathy indications. This limits the conclusions for individual non-dysthyroid optic neuropathy indications.

In summary, in this cohort of patients undergoing orbital apex decompression combined with intraorbital corticosteroid injection while demonstrating elevated CAS was associated with reductions in CAS and proptosis, while postoperative worsening was infrequent. These findings suggest that active disease alone should not automatically preclude surgery in carefully selected patients with disabling symptoms, although larger cohorts stratified by surgical indication are needed before broader use can be recommended.

## Data Availability

The datasets generated and analyzed during the current study are not publicly available because they contain protected health information. De-identified data are available from the corresponding author on reasonable request, subject to institutional review board approval and a data use agreement.
